# Structural and Functional Characterization of the Newly Designed Antimicrobial Peptide Crabrolin21

**DOI:** 10.3390/membranes13030365

**Published:** 2023-03-22

**Authors:** Francesca Cantini, Paola Giannì, Sara Bobone, Cassandra Troiano, Hugo van Ingen, Renato Massoud, Nicoletta Perini, Luciana Migliore, Philippe Savarin, Charles Sanders, Lorenzo Stella, Marco Sette

**Affiliations:** 1 Magnetic Resonance Center (CERM), University of Florence, Sesto Fiorentino, 50019 Firenze, Italy; 2 Department of Chemistry, University of Florence, Sesto Fiorentino, 50019 Firenze, Italy; 3 Department of Chemical Sciences and Technology, University of Rome Tor Vergata, 00133 Rome, Italy; 4 Fondazione G.I.M.EM.A.-Franco Mandelli Onlus, 00182 Rome, Italy; 5 NMR Spectroscopy, Bijvoet Center for Biomolecular Research, Utrecht University, Padualaan 8, 3584 CH Utrecht, The Netherlands; 6 Department of Experimental Medicine, University of Rome Tor Vergata, Viale della Ricerca Scientifica, 00133 Rome, Italy; 7 Department of Biology, University of Rome Tor Vergata, 00133 Rome, Italy; 8 eCampus University, 22060 Novedrate, Italy; 9 Chemistry Structures Properties of Biomaterials and Therapeutic Agents Laboratory (CSPBAT), Nanomédecine Biomarqueurs Détection Team (NBD), Sorbonne Paris Nord University, The National Center for Scientific Research (CNRS), UMR 7244, 74 Rue Marcel Cachin, CEDEX, 93017 Bobigny, France; 10 Department of Biochemistry, Vanderbilt University School of Medicine, Nashville, TN 37232, USA; 11 Center for Structural Biology, Vanderbilt University School of Medicine, Nashville, TN 37232, USA; 12 Department of Medicine, Vanderbilt University School of Medicine, Nashville, TN 37232, USA

**Keywords:** antimicrobial resistance, membrane specificity, NMR spectroscopy, CD spectroscopy

## Abstract

(1) Background: antimicrobial resistance is becoming a dramatic problem for public health, and the design of new antimicrobial agents is an active research area. (2) Methods: based on our previous work, we designed an improved version of the crabrolin peptide and characterized its functional and structural properties with a wide range of techniques. (3) Results: the newly designed peptide, crabrolin21, is much more active than the previous ones and shows specific selectivity towards bacterial cells. (4) Conclusions: crabrolin21 shows interesting properties and deserves further studies.

## 1. Introduction

Antimicrobial resistance is a global and urgent health problem [1]. The increase in infections due to multidrug-resistant (MDR) bacteria requires the development of a new strategy to overcome this major health issue [2,3]. Within this framework, antimicrobial peptides (AMPs) are a promising possible solution. They are a class of natural molecules produced by all organisms (including humans) to fight microbial invasions. The general mode of action of most AMPs is not specific: perturbation of the permeability of bacterial membranes, peptide internalization followed by interaction with intracellular targets, and activation or modulation of the host immune response have been reported for the different AMPs [4,5,6,7,8,9]. Some AMPs are able to induce lipid phase separation. Others accelerate movements transversal to the membrane plane, referred to “flip-flop” [10]. The sequence of each peptide and factors such as the length of the peptide, the presence of hydrophilic residues at the membrane/water interface, or the presence of charged residues in the center of the peptide sequence determine the specific mechanism of action.

Antimicrobial activity based on the disruption of the membrane’s architecture occurs via electrostatic attraction followed by perturbation of the negatively charged bacterial membranes, leading to an increase in permeability [7]. On the contrary, the outer layer of eukaryotic membranes contains zwitterionic (mostly neutral) lipids, and this explains the different selectivity of AMPs. Because AMPs do not have a specific molecular target, the emergence of new resistant bacterial species is unlikely, and for this reason, they are considered a promising alternative to classical antibiotics, which both induce specific resistance and eradicate the gut flora, thus canceling the healthy effects of intestinal microbiota in preventing pathogenic bacteria from establishing dominance [11,12]. 

In this respect, an ideal antimicrobial peptide would have the ability to destroy bacterial membranes of multiple bacterial species as well as low toxicity for human cells, and for this purpose, the action of natural antimicrobial peptides can be improved by modifying the sequence.

We previously studied an antimicrobial peptide, named crabrolin, obtained from *Vespa crabro* [13]. This peptide is 13 amino acids long (FLPLILRKIVTAL-NH_2_), has a net positive charge of +2 at neutral pH, and shows little antimicrobial activity [14]. In amphiphilic solvents that mimic lipid membranes, it assumes an amphipathic helical conformation [15] as well as many other AMPs. Replacement of some neutral amino acids with positively charged ones (FLPKILRKIVRAL-NH_2_; crabrolin Plus) improves antimicrobial activity, maintaining the amphipathic helical propensity of the peptide, whereas a peptide in which the charge is diminished (FLPLILFWIVTAL-NH_2_; crabrolin Minus) displays no activity [14]. Furthermore, crabrolin Plus, in contrast to the wild-type peptide, binds to isolated lipopolysaccharide, altering its micellar state [16]. 

Following these previous studies, we designed a new peptide, crabrolin21, that contains extra residues at the C-terminal region and is also predicted to form an amphipathic helix.

This peptide was studied by means of both microbiological assays showing its improved antibacterial activity and spectroscopic techniques (CD and NMR) using liposomes mimicking prokaryotic or eukaryotic cell membranes, which indicates it does form an amphipathic helix and can selectively perturb bacterial membranes. Finally, specific interaction with lipopolysaccharide suggests that the peptide may have a multi-purpose usage. 

## 2. Materials and Methods

The peptide (FLPKILRKIVRALAKVGIKVA-NH_2_), named crabrolin21 hereafter, was obtained from Proteogenix at 98% purity. 

1,2-Dimyristoyl-*sn*-Glycero-3-Phosphocholine (DMPC), 1,2-Dimyristoyl-*sn*-glycero-3-phosphorylglycerol (DMPG), 1,2-Dihexanoyl-*sn*-Glycero-3-Phosphocholine (DHPC), 1-palmitoyl-2-oleoyl-sn-glycero-3-phosphoethanolamine (POPE), and 1-palmitoyl-2-oleoyl-sn-glycero-3-phospho-(1’-rac-glycerol) (POPG) were obtained from Avanti Polar Lipids, and their purity was >99%. Lipopolysaccharides from *Escherichia coli* O111:B4 (LPS) were purchased from Sigma-Aldrich (Milan, Italy).

### 2.1. Antimicrobial Susceptibility Tests

To evaluate the MIC (Minimal Inhibitory Concentration) of the antimicrobial peptides, the broth dilution method was performed according to the standard procedure by Wiegand and colleagues [17]. As in a previous study [4], the peptides were tested at an initial concentration of 600 μM. 

Precisely 100 μL of 2× Muller Hinton Broth (MHB) liquid was dispensed into each well of the microtiter plates, and 100 μL of the initial 2× peptide solution (1200 μg/mL) was added into the wells of plate column #1. Then, 100 μL were taken away from column #1 and added to column #2 to obtain a 1:2 serial dilution; this procedure was repeated up to column #6. From this last column, 100 μL were taken away and discarded. Next, 5 μL of each initial bacterial inoculum was then added from columns #1 to #6 to obtain the final density of 1 x10^5^ CFU/well. Columns #7 and #8 of the multiwell plates were used for controls: column #7 was a positive control, containing MHB and bacteria, while column #8 was the broth sterility control, containing only MHB and the tested peptide. Each test was performed in triplicate, each consisting of one row in the plate, and the experiment was performed three times. Furthermore, the test to evaluate the potential toxic effect of the crabrolin Minus suspension buffer, i.e., 10 mM potassium phosphate buffer solution (K_3_PO_4_), was repeated three times (pH 6.5). The plates were covered and incubated overnight at 37 °C. The growth in each well was visually detected as turbidity in the well. 

### 2.2. Liposome Preparation

Large unilamellar vesicles (LUVs) composed of POPE/POPG (7:3 molar ratio) or POPC/cholesterol (1:1 molar ratio) were prepared by dissolving the lipids in a 1:1 (*v*/*v*) CHCl_3_/MeOH solution. The solvent was then evaporated in a rotary vacuum system until a thin film was formed. Complete evaporation was ensured by applying a rotary vacuum pump for at least 2 h. The lipid film was hydrated with a 30 mM 5,6-carboxyfluorescein (CF) solution, titrated to pH 7.4 with NaOH, containing 10 mM phosphate buffer and 80 mM NaCl to make it isotonic to the dilution buffer (phosphate buffer 10 mM, pH 7.4, NaCl 140 mM, 270 mOsm). We have previously shown that at 30 mM, CF is ~80% self-quenched [18].

The liposome suspension was vigorously stirred and, after 10 freeze-and-thaw cycles, was extruded through two stacked polycarbonate membranes with pores of nominal diameter 100 nm 31 times. Liposomes were separated from an unencapsulated dye by gel filtration on a 40 cm Sephadex G-50 column (SIGMA Life Science, Merck, Darmstadt, Germany). The final lipid concentration was determined by the Stewart phospholipids assay [19]. Liposomes for CD experiments were prepared following a similar procedure but were hydrated with a phosphate buffer (10 mM, pH 6.5) and extruded through polycarbonate membranes with pores of nominal diameter 50 nm.

### 2.3. CD Experiments

Measurements were performed on a Jasco J-1500 spectropolarimeter (JASCO Corporation, Tokyo, Japan). The peptide (11 μM concentration) was titrated with increasing amounts of lipid vesicles. The following experimental conditions were used: optical path length 0.1 cm; interval scan 260–180 nm; sampling interval 0.2 nm; scanning speed 50 nm/min; and bandwidth 2 nm. Each measurement was the result of 8 accumulations. Every spectrum was analyzed after subtraction of the liposome signal at the corresponding concentration. 

Assuming a two-state equilibrium between membrane-bound and unbound peptide, and considering that the CD is an additive, linear signal, the molar ellipticity at a fixed wavelength (197 nm in our case) at a given lipid concentration [L] can be expressed as a function of the fraction of membrane-bound peptides, $f_{b}$ [20]:

(1) $$\theta \left(\left[L\right]\right)=f_{b}\left(\left[L\right]\right)\times \theta_{b}+\left(1-f_{b}\left(\left[L\right]\right)\right)\theta_{w}$$

where $\theta_{w}$ and $\theta_{b}$ refer to the signal of completely bound and free peptides, respectively. 

Assuming that peptide/membrane association can be described by an ideal partition equilibrium, the fraction of membrane-bound peptide depends on lipid concentration as follows [Stella 2004]:

(2) $$f_{b}\left(\left[L\right]\right)=\frac{K_{P}\frac{\left[L\right]}{\left[W\right]}}{1+K_{P}\frac{\left[L\right]}{\left[W\right]}}$$

where $K_{P}$ is the partition constant and [W] is the molar concentration of pure water.

$\theta_{w}$ is directly measured for the peptide in the absence of membranes. By contrast, reaching a condition of a completely bound peptide to measure $\theta_{b}$ is often impossible for practical reasons. This was the case in the present study because lipid concentrations above those used here cause significant CD artifacts due to light scattering. Therefore, the data for POPG/POPE lipids were fit by combining the two above equations:

(3) $$\theta \left(\left[L\right]\right)=\theta_{w}+\left(\theta_{b}-\theta_{w}\right)\frac{K_{P}\frac{\left[L\right]}{\left[W\right]}}{1+K_{P}\frac{\left[L\right]}{\left[W\right]}}$$

Both $K_{P}$ and $\theta_{b}$ were left as free-fitting parameters. In the case of POPC/cholesterol lipids, saturation was too far from being achieved to allow the estimate of $\theta_{b}$ from a fit of the data. However, it is reasonable to assume that the lipid composition influences peptide/membrane affinity but not the secondary structure of the peptide. Therefore, the same $\theta_{b}$ value was used for analyzing both datasets (POPG/POPE and POPC/cholesterol). The results are reported as a fraction of membrane-bound peptide, determined according to Equation (1).

Spectra of the peptide dissolved in MeOH or TFE were acquired under the same experimental conditions.

### 2.4. Membrane-Perturbing Activity Experiments

Studies on vesicle release kinetics were performed on an Infinite M200 PRO plate reader (Tecan, Grödig, Austria). The temperature was maintained at 25 °C for all experiments. The percentage of peptide-induced CF leakage was measured by following the fluorescence signal at λ_ex_ = 490 nm and λ_em_ = 520 nm, with an excitation bandwidth of 9 nm and emission bandwidth of 20 nm and a spacing of 30 s. Measurements were repeated three times. Total leakage was obtained by adding Triton X-100 (SIGMA Life Science, Merck, Darmstadt, Germany) at a 1 mM final concentration. The fraction of peptide-induced leakage was determined as

(4) $$Released\;fraction=\frac{F-F_{0}}{F_{100}-F_{0}}$$

where *F* is the fluorescence signal recorded 20 min after the addition of the peptide, *F*_0_ is the initial fluorescence of vesicles, and *F*_100_ is the fluorescence obtained after the addition of Triton X-100. The experiment was performed with 50 µM total lipid concentration and 1.5 µM peptide.

### 2.5. Hemolytic Activity Assay

Blood was washed six times with 5 mM HEPES, pH 7.3, 150 mM NaCl (buffer E), and resuspended in the same buffer. After this step, RBC density was measured with an automated hematology analyzer Sysmex XE-2100 (TOA Medical Electronics, Kobe, Japan). Aliquots of the red blood cells (RBC) suspension, diluted in buffer E at a final concentration of 5 × 10^8^ cells/mL, were incubated with nine different concentrations of crabrolin21 (from 0.4 μM to 100 μM) at 37 °C for 30 min. Negative control was obtained by suspending RBCs in buffer E without any peptide. Total hemolysis (positive control) was obtained by suspending RBCs in distilled water overnight (osmotic shock).

The hemolytic activity of crabrolin21 was measured on human RBCs following the previously published protocol [21]. Briefly, after peptide incubation, the RBC samples were centrifuged in an SL8R Thermo Scientific (Waltham, MA, USA) centrifuge for 10 min at 1100× *g*, and the absorbance (*Abs*) of the supernatant was measured on a Cary-UV 100 Scan spectrophotometer (Varian, Middelburg, Netherlands) at 540 nm using 1 cm pathlength cuvettes. The hemoglobin release (i.e., normalized absorbance) was calculated as follows:

(5) $$\;hemoglobin\;release\;\left(\%\right)=\frac{Abs-Abs_{NC}}{Abs_{PC}-Abs_{NC}}$$

(where the subscript *NC* and *PC* denotes the positive control samples). Datapoints were interpolated with a phenomenological Hill equation:

(6) $$\;\;\;\;\;\;\;\;y=\frac{100}{1+{\left(\frac{K}{x}\right)}^{n}}$$

where *x* denotes the peptide concentration. The parameter *K* is the peptide concentration corresponding to half RBCs hemolysis, whereas the parameter *n* is the Hill coefficient, varying the steepness of the curve.

### 2.6. NMR Spectroscopy

Bicelles were prepared using a buffer solution containing 10 mM potassium phosphate at pH 6.5 and mixing a buffered solution of DMPC and DMPG with a buffered solution of DHPC, according to the reported literature [22]. The final value of q was 0.21, where q represents the ratio of moles of lipid (DMPC + DMPG) to moles of detergent (DHPC).

NMR spectra were collected on a Bruker Avance III HD spectrometer (Bruker Biospin, Ettlingen, Germany) operating at 900 MHz. Experiments for the sample in TFE were recorded at 300 K and consisted of a clean-TOCSY (80 ms mixing time) and a NOESY (150 ms mixing time).

Experiments with non-deuterated bicelles were recorded at 308 K and consisted of TOCSY (at 50 and 80 ms) and NOESY spectra (at 60 and 80 ms of mixing times to avoid spin-diffusion). The pulse sequences contained a 90-degree PC9 reading-shaped pulse [23] centered on the amino region to suppress the bulk hydrogen signals from the lipids. Thus, the analysis was conducted without using the aliphatic region of the spectra. Spectra were referenced to external DSS.

Phosphorous 31 spectra were collected on a Bruker Avance spectrometer operating at 400 MHz by using an inverse-gated one-pulse experiment. 

Data were processed with NMRPipe [24] under the NMRbox tool [25], and data analysis was performed with NMRFAM-SPARKY [26] or CARA [27]. Structure calculation was performed with CYANA 3.98 [28]. Figures were drawn with CHIMERA software [29].

## 3. Results

In light of the previous results [16], we were interested in improving the antimicrobial activity of crabrolin Plus and designed a longer peptide, crabrolin21, that was predicted to maintain the amphipathic character of the previous one. 

The factors believed to be important for antimicrobial activity are the peptide hydrophobicity, the presence of positively charged residues, an amphipathic nature that separates basic from hydrophobic residues, and the tendency to form an alpha-helix [7]. Within this frame, the new peptide was designed, starting from the structure of crabrolin Plus, adding eight extra residues at the C-terminal tail in which two positively charged residues are included, properly alternated by hydrophobic residues. The net charge increases by two units and the tendency to form an amphipathic helix has been evaluated by CHIMERA software, version 1.16 (University of California, San Francisco, USA) [29].

### 3.1. Antimicrobial Assays

The comparison of the antibacterial activity of crabrolin WT, crabrolin Minus, and crabrolin Plus vs. crabrolin21 is reported in Table 1.

The previous results indicated that crabrolin WT had a moderate effect on Gram-positive and Gram-negative species and that crabrolin Plus is two to eight times more active. The present tests show that crabrolin21 is much more active than crabrolin Plus, considering both the MIC values (with a reduction of the values from 6 times to almost 50 times depending on the bacterial species) and across most of the tested microbial spectrum. In particular, crabrolin21 is the only peptide that shows antimicrobial activity against *P. aeruginosa* ATCC 27853. Unfortunately, it has no activity against *P. mirabilis* CCUG 26767.

### 3.2. Membrane-Perturbing Activity

**Figure 1:**
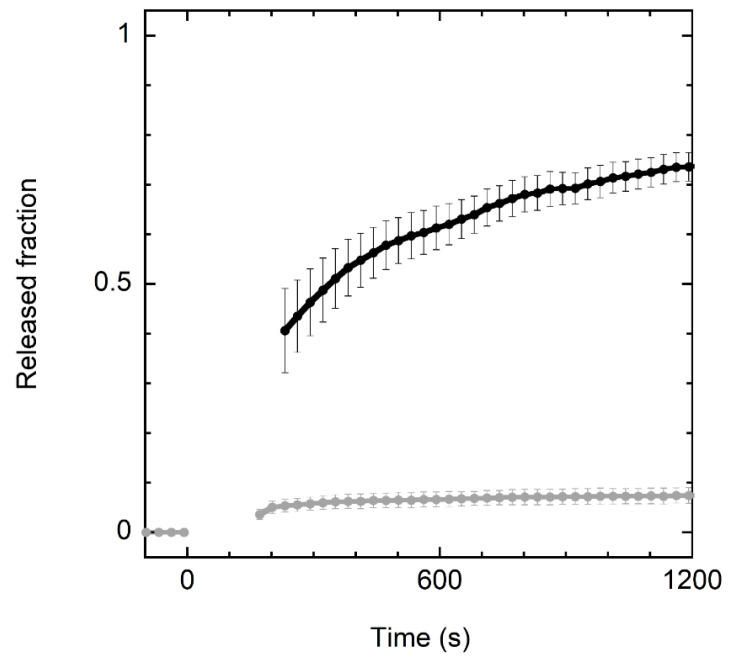
Kinetics of CF release from vesicles after addition of crabrolin21. Kinetics of CF release after addition of crabrolin21 to POPE/POPG (7:3 molar ratio) or POPC/cholesterol vesicles (1:1 molar ratio) (black and gray lines, respectively). Liposome concentration: 50 μM. Crabrolin21 concentration: 1.5 μM. Measurements were performed at 298 K and pH 7.4. Standard errors over three measurements are reported.

Experiments with model membranes indicated that crabrolin21 activity is based on its ability to perturb the permeability of lipid bilayers. The addition of crabrolin21 to a liposome suspension was able to cause the leakage of a fluorophore entrapped inside the vesicles (Figure 1). 

Interestingly, crabrolin21 peptide shows significant selectivity for bilayers mimicking the composition of bacterial membranes (POPE/POPG, 7:3 molar ratio) over those mimicking eukaryotic membranes (POPC/cholesterol, 1:1 molar ratio). This finding is promising in view of potential therapeutic applications.

### 3.3. Secondary Structure of the Peptide: CD Experiments

**Figure 2:**
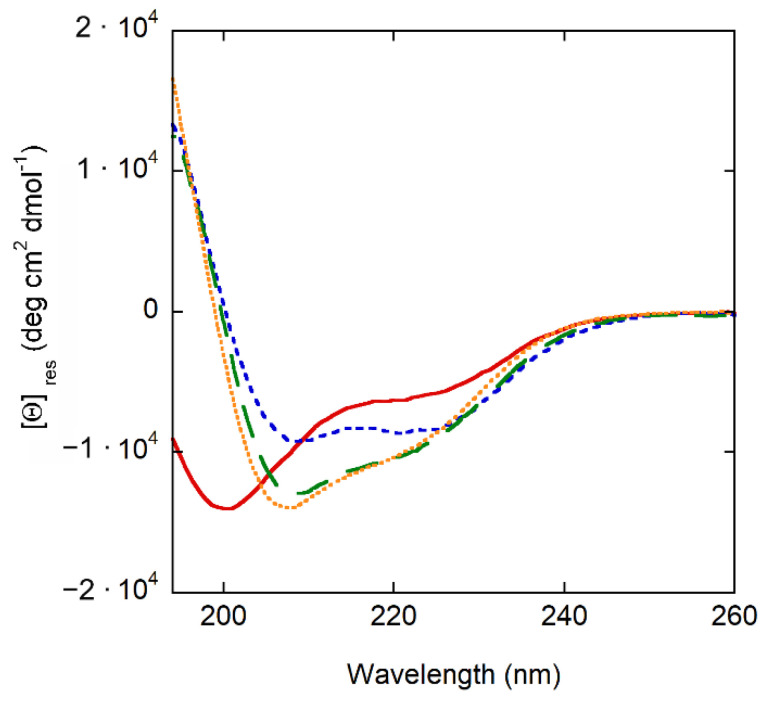
Secondary structure of crabrolin21 in different systems. Far UV circular dichroism spectra of crabrolin21 in aqueous buffer solution (solid red line), methanol (green dashed line), TFE (orange dotted line), and in the presence of POPE/POPG (7:3 molar ratio) at 500 µM total lipid concentration in phosphate buffer (10 mM, pH 6.5) (blue short-dashed line). Crabrolin21 concentration: 11 μM. Measurements were performed at 298 K and pH 7.4.

CD experiments show that crabrolin21 is largely unstructured in an aqueous solution; it becomes partially helical in solvents of lower polarity (TFE and methanol) and mostly helical when membrane-bound (Figure 2).

### 3.4. Peptide–Membrane Association

**Figure 3:**
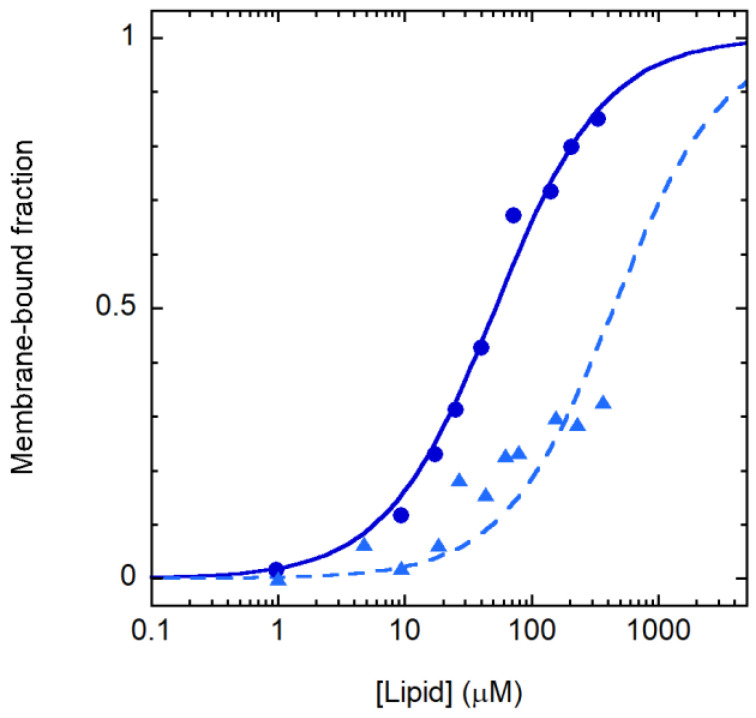
Crabrolin21 binding to membranes. Crabrolin21/membrane association followed through the peptide CD signal at 197 nm for POPE/POPG (7:3 molar ratio) or POPC/cholesterol vesicles (1:1 molar ratio) (blue circles and light blue triangles, respectively). Crabrolin21 concentration: 11 μM. Measurements were performed at 298 K and pH 7.4.

The variation in the CD spectrum caused by membrane association was exploited to follow partitioning between the aqueous and lipid phase. The resulting binding curves are reported in Figure 3 and indicate that the selectivity observed in the leakage experiments is caused by differential membrane binding to POPE/POPG and POPC/cholesterol vesicles. The partition constants [30] for the two membrane types were (1.1 ± 0.1) 10^6^ and (0.13 ± 0.02) 10^6^, respectively, in favor of the membrane.

### 3.5. Toxicity Assay

**Figure 4:**
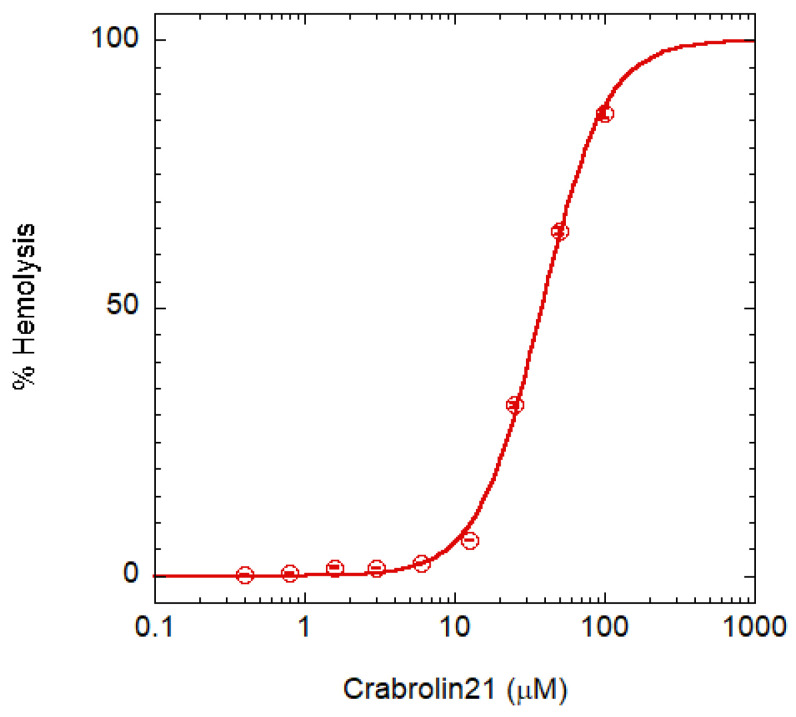
RBC lysis assay. The assay is based on hemoglobin release; circles are the experimental data points, while the continuous line is a guide for the eye. Error bars indicate the interval of duplicate measurements.

Toxicity was assayed on red blood cells (RBCs) Figure 4. A crabrolin concentration higher than 100 μM is required for complete hemolysis (HC100%). Taking into account that the MIC is 4 μM for several bacteria, the peptide has a therapeutic index (i.e., the ratio between HC100% and MIC) >25. Even though this commonly used assay of AMP selectivity has some limitations [21], crabrolin21 selectivity is in line with those of most AMPs [31].

A possible strategy to reduce the hemolysis would be the removal of amidation at the C-terminal position, but this modification generally produces an increase in the MIC [32].

### 3.6. NMR Spectroscopy

To better understand the structural features of the new peptide, solution 2D NMR spectra were obtained using negatively charged isotropic bicelles in a (DMPC + DMPG)/DHPC q ratio of 0.21. In the absence of bicelles, crabrolin21 at the same experimental conditions shows an NMR spectrum typical of an unstructured peptide, i.e., little dispersion of resonances, small linewidth, and lack of intermolecular NOEs (data not shown). In contrast, in the presence of bicelles, resonances broaden, and more NOEs are present in the spectrum at a short mixing time, suggesting that the peptide binds to the bicelles and assumes their correlation time. Analysis of TOCSY and NOESY spectra allowed us to assign the resonances for the backbone and a few side chains. Because the spectra were obtained using protonated bicelles, the peptidic aliphatic region was obscured by the huge signals of the phospholipids, thus hampering the measurement of NOEs in this region for use in calculating a tertiary structure. Furthermore, at this q-ratio, very few side chains were observed in the TOCSY spectrum at 50 ms (Figure S1 in the Supplementary Materials) or 80 ms, presumably, for the correlation time of the system; thus, assignment relies on the HN-HN and Hα-HN connectivities. Cross-peaks for the residues from 1 to 6 were not observed in TOCSY or NOESY spectra, suggesting conformational disorder for this region. The remaining residues were successfully assigned, and the secondary structure of crabrolin21 was estimated by using the Chemical Shift Index of the alpha protons [33], a reliable indicator of the secondary structure. In this approach, a contiguous series of chemical shift values higher than the random coil’s chemical shift values suggests the presence of beta strands, while a contiguous series of values lower than the random coil’s chemical shift values suggests the presence of an alpha-helix. The Chemical Shift Index of crabrolin21 (Figure S2 in the Supplementary Materials) shows that the peptide forms an alpha-helix from Arg7 to Lys15. Residues 16 and 20 possess a chemical shift higher than the random coil values, but because they do not belong to a contiguous series of chemical shifts, they are not assumed to belong to the beta-strand structure. 

**Figure 5:**
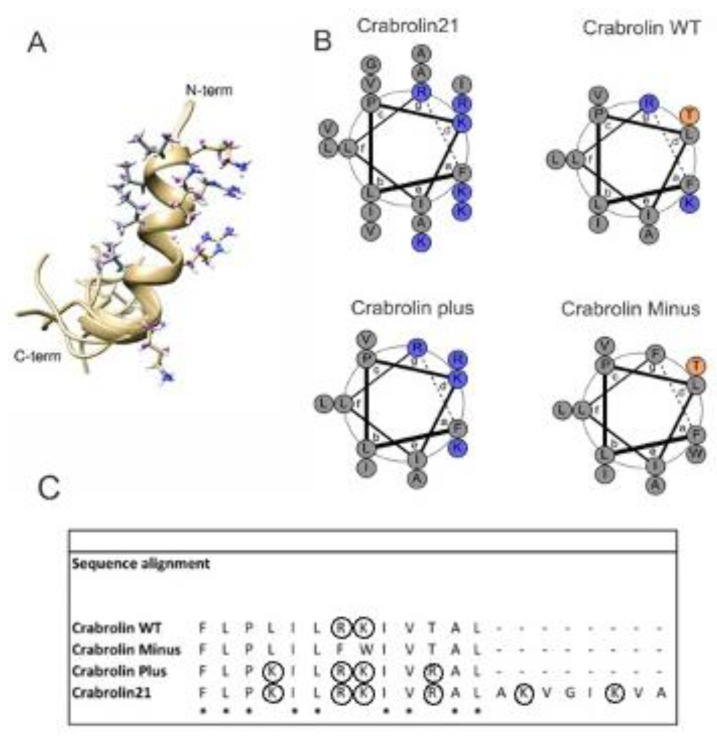
Solution structure of crabrolin21 and comparison with previous crabrolin peptides. (**A**): NMR solution structure of crabrolin21 peptide in TFE. The family of 10 conformers was obtained by torsion angle dynamics by using the CYANA 3.98 software [28]. The side chains of charged amino acids (arginines and lysines) of the first conformer are shown on the right side, and the hydrophobic side chains are shown on the left side. (**B**): Helical wheels of crabrolin21, crabrolin WT, crabrolin Minus, and crabrolin Plus with the charged residues colored in blue. (**C**): sequence alignment of crabrolin peptides. Conserved residues are marked with an asterisk, and charged residues are circled. Helical wheels were obtained with DrawCoil [35].

NMR spectra were conducted in the presence of TFE to check the helical propensity of the peptide. The analysis of the ^1^H-^1^H NOESY spectrum confirms the presence of a well-defined helix between Lys4 and Val16, in good agreement with the results obtained in the presence of bicelles. Several medium C_α_H/NH (i to i + 2 and i to i + 3) and C_α_H/C_β_H (i to i + 3) NOEs, diagnostic of the helical conformation, are indeed detected for these residues (Figure S3 in the Supplementary Materials). The solution structure of the peptide is reported in Figure 5A and shows that it forms a helical region in the middle of its sequence and maintains an amphipathic structure. The conformational and energetic analysis, together with selected quality parameters from PSVS [34] software of the final family of peptide conformations, is reported in Table S1 in the Supplementary Materials. 

Ramachandran plot and NOEs restrains table are reported in Figures S4 and S5 in the Supplementary Materials.

A helical-wheel comparison of crabrolin21 with previous crabrolin peptides (crabrolin WT, crabrolin Plus, and crabrolin Minus) is reported in Figure 5B, and their sequence alignment is in Figure 5C. Analysis of the peptides indicates that all the charged residues are located in the same region of the peptide and suggests that other residues can be modified as well. On the other hand, the hydrophobic surface could also be modified, bearing in mind that variants of specific residues, such as Pro3, may affect the hemolysis of the peptide [36].

**Figure 6:**
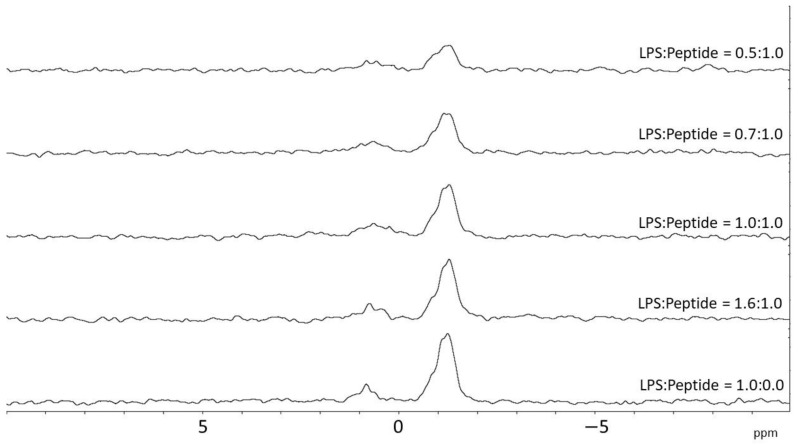
Phosphorous NMR of lipopolysaccharide from *E. coli* in the presence of increasing amounts of crabrolin21. LPS from *E.coli* was titrated with increasing amounts of crabrolin21. Measurements were performed at 298 K and pH 6.5.

To further investigate the details of the interaction of crabrolin21 with the bacterial membrane, a ^31^P NMR titration of lipopolysaccharide micelles from *E. coli* was performed by adding increasing amounts of the peptide as previously reported for the other crabrolin peptides [14]. The results, reported in Figure 6, indicate an interaction of crabrolin21 with this key component of the membranes of Gram-negative bacteria.

## 4. Discussion

We have analyzed the structural and functional properties of a new peptide, named crabrolin21, derived from the crabrolin peptide. Crabrolin (FLPLILRKIVTAL-NH_2_) has a low antimicrobial activity [14] and, in a previous study, we showed that the replacement of some of the uncharged residues by cationic ones (FLPKILRKIVRAL-NH_2_) has a positive effect on the antimicrobial activity and the resulting peptide possesses an amphipathic structure [14]. Following this line of reasoning and using the previous structure of the more active peptide as a template, we designed a new, longer peptide containing additional amino acids in the C-terminal region of modified crabrolin while also trying to maintain the amphipathic properties of the molecule. The new peptide (FLPKILRKIVRALAKVGIKVA-NH_2_), named crabrolin21, is very active on bacteria of high clinical relevance, including both Gram-negative and Gram-positive species. Indeed, *E. coli* is frequently isolated from many human infections, often exhibiting antibiotic-resistance traits; *K. pneumoniae* is a common nosocomial pathogen, considered a serious risk for human health due to the emergence and spreading of multi- or extremely drug-resistant strains; *P. aeruginosa* is a widespread cause of infections in immuno-compromised individuals as well as in cystic fibrosis patients and is often endowed with a marked resistance to antibiotics; *S. enterica* Typhimurium is a frequent cause of diarrheal diseases that can pose risks for children, elderly, and immuno-compromised patients; finally, *S. aureus* has become a major threat in hospital and outpatient settings due to its resistance to beta-lactams. Many of the strains utilized in these tests are employed in quality control procedures for antibiotic determination testing (*E. coli*, *P. aeruginosa*, *S. enterica* Typhimurium, *B. subtilis*, and *S. aureus*). Hemolytic activity assays showed that the peptide has a therapeutic index higher than 25. 

Circular dichroism and permeability studies indicated that crabrolin21 is very active at the level of the plasma membrane, and its effect is specific for membranes mimicking the bacterial plasma membranes rather than eukaryotic membranes, an important feature for clinical application. Furthermore, the hydrophobic membrane environment induces the folding of the peptide that converts from a random coil to a helical structure. For Gram-negative bacteria, we also observed the binding of the peptide to lipopolysaccharide, which could help to destabilize the bacterial membrane. 

NMR studies in an amphiphilic solvent, as well as in isotropic bicelles mimicking the bacterial membrane, indicate that the peptide mainly assumes a helical structure and maintains an amphipathic character. Comparison with previous crabrolins shows that the location of hydrophobic and hydrophilic residues is maintained and that the newly added residues confer a more pronounced electrostatic contribution without altering the amphipathic nature of the molecule. Such results are important in light of clinical application and pave the way for designing a still more active peptide by modulating its amino acid nature or position.

Finally, the ability of crabrolin21 to bind with LPS suggests that the interaction with Gram-negative bacteria could be mediated by this molecule and, because the release of LPS aggregates during bacterial infection may activate uncontrolled inflammatory response [37], crabrolin21 could be useful to alleviate this relevant clinical issue. 

## Figures and Tables

**Table 1:** Minimal inhibitory concentration (MIC) for crabrolin21 and comparison with previous crabrolins (crabrolin WT, crabrolin Plus, and crabrolin Minus). (^a^) Values are from [14] and (^b^) no inhibition (n.i.) tested until 600 μM.

	Crabrolin WT ^a^	Crabrolin Minus ^a^	Crabrolin Plus ^a^	Crabrolin21	Buffer ^b^
Minimal Inhibitory Concentration (µM)					
Gram-negative bacteria					
*Escherichia coli* ATCC 25922	200	>383	24	4	n.i.
*Pseudomonas* *aeruginosa* ATCC 27853	>400	>383	> 383	16	n.i.
*Proteus mirabilis* CCUG 26767	>400	>383	>383	>257	n.i.
*Salmonella enterica* Typhimurium LT2	200	>383	48	8	n.i.
*Klebsiella* *pneumoniae* ATCC 13883	>400	>383	48	4	n.i.
Gram-positive bacteria					
*Bacillus subtilis* ATCC 6633	200	>383	48	4	n.i.
*Staphylococcus* *aureus* ATCC 29213	200	>383	190	4	n.i.

## Data Availability

Not applicable.

## Supplementary Materials

The following are available online at https://www.mdpi.com/article/10.3390/membranes13030365/s1, Figure S1: 2D-NMR spectra of crabrolin21 in different systems; Figure S2: Chemical Shift Index of crabrolin21 in bicelles; Figure S3: Observed NOEs for crabrolin21 in TFE; Figure S4: Ramachandran plot for crabrolin21 in TFE; and Figure S5: NMR distance constraints for crabrolin21 in TFE. Table S1: Summary of restraints and structure quality factors of the determined family of conformations for crabrolin21 in TFE.

## References

[1] Murray C.J., Ikuta K.S., Sharara F., Swetschinski L., Aguilar G.R., Gray A., Han C., Bisignano C., Rao P., Wool E. (2022). Global burden of bacterial antimicrobial resistance in 2019: A systematic analysis. Lancet.

[2] Ibrahim M.E., Bilal N.E., Hamid M. (2013). Increased multi-drug resistant Escherichia coli from hospitals in Khartoum state, Sudan. Afr. Health Sci..

[3] Mulani M.S., Kamble E.E., Kumkar S.N., Tawre M.S., Pardesi K.R. (2019). Emerging Strategies to Combat ESKAPE Pathogens in the Era of Antimicrobial Resistance: A Review. Front. Microbiol..

[4] Steinstraesser L., Kraneburg U., Jacobsen F., Al-Benna S. (2011). Host defense peptides and their antimicrobial-immunomodulatory duality. Immunobiology.

[5] Yeung A.T.Y., Gellatly S.L., Hancock R.E.W. (2011). Multifunctional cationic host defence peptides and their clinical applications. Cell. Mol. Life Sci..

[6] Hancock R.E.W., Haney E.F., Gill E.E. (2016). The immunology of host defence peptides: Beyond antimicrobial activity. Nat. Rev. Immunol..

[7] Nguyen L.T., Haney E.F., Vogel H.J. (2011). The expanding scope of antimicrobial peptide structures and their modes of action. Trends Biotechnol..

[8] Epand R.M., Vogel H.J. (1999). Diversity of antimicrobial peptides and their mechanisms of action. Biochim. Biophys. Acta.

[9] Nicolas P. (2009). Multifunctional host defense peptides: Intracellular-targeting antimicrobial peptides. FEBS J..

[10] McHenry A.J., Sciacca M.F., Brender J.R., Ramamoorthy A. (2012). Does cholesterol suppress the antimicrobial peptide induced disruption of lipid raft containing membranes?. Biochim. Biophys. Acta.

[11] Yu G., Baeder D.Y., Regoes R.R., Rolff J. (2018). Predicting drug resistance evolution: Insights from antimicrobial peptides and antibiotics. Proc. Biol. Sci..

[12] Ramos-Martín F., Herrera-León C., Antonietti V., Sonnet P., Sarazin C., D’Amelio N. (2020). Antimicrobial Peptide K11 Selectively Recognizes Bacterial Biomimetic Membranes and Acts by Twisting Their Bilayers. Pharmaceuticals.

[13] Argiolas A., Pisano J.J. (1984). Isolation and characterization of two new peptides, mastoparan C and crabrolin, from the venom of the European hornet, Vespa crabro. J. Biol. Chem..

[14] Aschi M., Perini N., Bouchemal N., Luzi C., Savarin P., Migliore L., Bozzi A., Sette M. (2019). Structural characterization and biological activity of Crabrolin peptide isoforms with different positive charge. Biochim. Biophys. Acta Biomembr..

[15] Aschi M., Bozzi A., Luzi C., Bouchemal N., Sette M. (2017). Crabrolin, a natural antimicrobial peptide: Structural properties. J. Pept. Sci..

[16] Cantini F., Luzi C., Bouchemal N., Savarin P., Bozzi A., Sette M. (2020). Effect of positive charges in the structural interaction of crabrolin isoforms with lipopolysaccharide. J. Pept. Sci..

[17] Wiegand I., Hilpert K., Hancock R.E.W. (2008). Agar and broth dilution methods to determine the minimal inhibitory concentration (MIC) of antimicrobial substances. Nat. Protoc..

[18] Orioni B., Bocchinfuso G., Kim J.Y., Palleschi A., Grande G., Bobone S., Park Y., Kim J.I., Hahm K.-S., Stella L. (2009). Membrane perturbation by the antimicrobial peptide PMAP-23: A fluorescence and molecular dynamics study. Biochim. Biophys. Acta Biomembr..

[19] Stewart J.C.M. (1980). Colorimetric determination of phospholipids with ammonium ferrothiocyanate. Anal. Biochem..

[20] Bocchinfuso G., Bobone S., Mazzuca C., Palleschi A., Stella L. (2011). Fluorescence spectroscopy and molecular dynamics simulations in studies on the mechanism of membrane destabilization by antimicrobial peptides. Cell. Mol. Life Sci..

[21] Savini F., Luca V., Bocedi A., Massoud R., Park Y., Mangoni M.L., Stella L. (2016). Cell-Density Dependence of Host-Defense Peptide Activity and Selectivity in the Presence of Host Cells. ACS Chem. Biol..

[22] Charles R., Sanders I.I., Schwonek J.P. Characterization of Magnetically Orientable Bilayers in Mixtures of Dihexanoylphosphatidylcholine and Dimyristoylphosphatidylcholine by Solid-State NMR. https://pubs.acs.org/doi/pdf/10.1021/bi00152a029.

[23] Kupce E., Freeman R. (1993). Polychromatic Selective Pulses. J. Magn. Reson. Ser. A.

[24] Delaglio F., Grzesiek S., Vuister G.W., Zhu G., Pfeifer J., Bax A. (1995). NMRPipe: A multidimensional spectral processing system based on UNIX pipes. J. Biomol. NMR.

[25] Maciejewski M.W., Schuyler A.D., Gryk M.R., Moraru I.I., Romero P.R., Ulrich E.L., Eghbalnia H.R., Livny M., Delaglio F., Hoch J.C. (2017). NMRbox: A Resource for Biomolecular NMR Computation. Biophys. J..

[26] Lee W., Tonelli M., Markley J.L. (2015). NMRFAM-SPARKY: Enhanced software for biomolecular NMR spectroscopy. Bioinformatics.

[27] Keller R. (2004). The Computer Aided Resonance Assignment Tutorial.

[28] Güntert P., Buchner L. (2015). Combined automated NOE assignment and structure calculation with CYANA. J. Biomol. NMR.

[29] Pettersen E.F., Goddard T.D., Huang C.C., Couch G.S., Greenblatt D.M., Meng E.C., Ferrin T.E. (2004). UCSF Chimera? A visualization system for exploratory research and analysis. J. Comput. Chem..

[30] Stella L., Mazzuca C., Venanzi M., Palleschi A., Didonè M., Formaggio F., Toniolo C., Pispisa B. (2004). Aggregation and Water-Membrane Partition as Major Determinants of the Activity of the Antibiotic Peptide Trichogin GA IV. Biophys. J..

[31] Bobone S., Stella L. (2019). Selectivity of Antimicrobial Peptides: A Complex Interplay of Multiple Equilibria. Adv. Exp. Med. Biol..

[32] Strandberg E., Tiltak D., Ieronimo M., Kanithasen N., Wadhwani P., Ulrich A.S. (2007). Influence of C-terminal amidation on the antimicrobial and hemolytic activities of cationic α-helical peptides. Pure Appl. Chem..

[33] Wishart D.S., Sykes B.D., Richards F.M. (1992). The chemical shift index: A fast and simple method for the assignment of protein secondary structure through NMR spectroscopy. Biochemistry.

[34] Bhattacharya A., Tejero R., Montelione G.T. (2006). Evaluating protein structures determined by structural genomics consortia. Proteins.

[35] Grigoryan G., Keating A.E. (2008). Structural specificity in coiled-coil interactions. Curr. Opin. Struct. Biol..

[36] Krishnakumari V., Nagaraj R. (2009). Antimicrobial and hemolytic activities of crabrolin, a 13-residue peptide from the venom of the European hornet, Vespa crabro, and its analogs. J. Pept. Res..

[37] Cohen J. (2002). The immunopathogenesis of sepsis. Nature.

