# Supplementary material for: Structural and Functional Characterization of the Newly Designed Antimicrobial Peptide Crabrolin21
Source: Membranes (Basel). 2023 Mar 22;13(3):365. doi: 10.3390/membranes13030365 (PMC10053045; doi:10.3390/membranes13030365)
Supplement: Supplementary file 1 [file membranes-13-00365-s001.zip › membranes-2227166-supplementary.pdf]

membranes

MDPI

Supplementary information

Structural and Functional Characterization of the Newly Designed Antimicrobial Peptide Crabrolin21

Francesca Cantini [1,2], Paola Giannì [3,4], Sara Bobone [3], Cassandra Troiano [3], Hugo van Ingen [5], Renato Massoud [6], Nicoletta Perini [7], Luciana Migliore [7,8], Philippe Savarin [9], Charles Sanders [10,11,12], Lorenzo Stella [3] and Marco Sette [3,9,*]

[1] Magnetic Resonance Center (CERM), University of Florence, Sesto Fiorentino, 50019 Firenze, Italy
[2] Department of Chemistry, University of Florence, Sesto Fiorentino, 50019 Firenze, Italy
[3] Department of Chemical Sciences and Technology, University of Rome Tor Vergata, 00133 Rome, Italy
[4] Fondazione G.I.M.EM.A.-Franco Mandelli Onlus, 00182 Rome, Italy
[5] NMR Spectroscopy, Bijvoet Center for Biomolecular Research, Utrecht University, Padualaan 8, 3584 CH Utrecht, The Netherlands
[6] Department of Experimental Medicine, University of Rome Tor Vergata, Viale della Ricerca Scientifica, 00133 Rome, Italy
[7] Department of Biology, University of Rome Tor Vergata, 00133 Rome, Italy
[8] eCampus University, 22060 Novedrate, Italy
[9] Chemistry Structures Properties of Biomaterials and Therapeutic Agents Laboratory (CSPBAT), Nanomédecine Biomarqueurs Détection Team (NBD), Sorbonne Paris Nord University, The National Center for Scientific Research (CNRS), UMR 7244, 74 Rue Marcel Cachin, CEDEX, 93017 Bobigny, France
[10] Department of Biochemistry, Vanderbilt University School of Medicine, Nashville, TN 37232, USA
[11] Center for Structural Biology, Vanderbilt University School of Medicine, Nashville, TN 37232, USA
[12] Department of Medicine, Vanderbilt University School of Medicine, Nashville, TN 37232, USA
* Correspondence: sette@uniroma2.it; Tel.: +39-067-259-4424

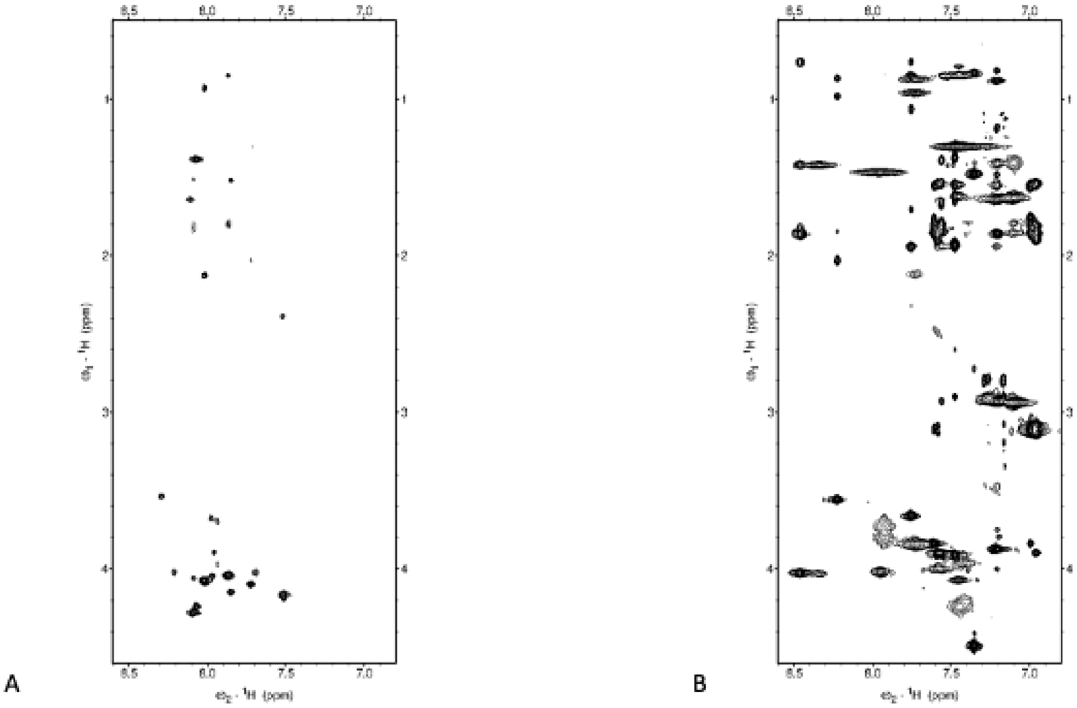

Figure S1. 2D-NMR spectra of crabrolin21 in different systems. Left panel: amidic vs. aliphatic region of the TOCSY spectrum in bicelles collected at 50 ms mixing time. Measurements were performed at 308 K and pH 6.5. Right panel: amidic vs. aliphatic region of the TOCSY spectrum in TFE collected at 80 ms mixing time. Measurements were performed at 300 K.

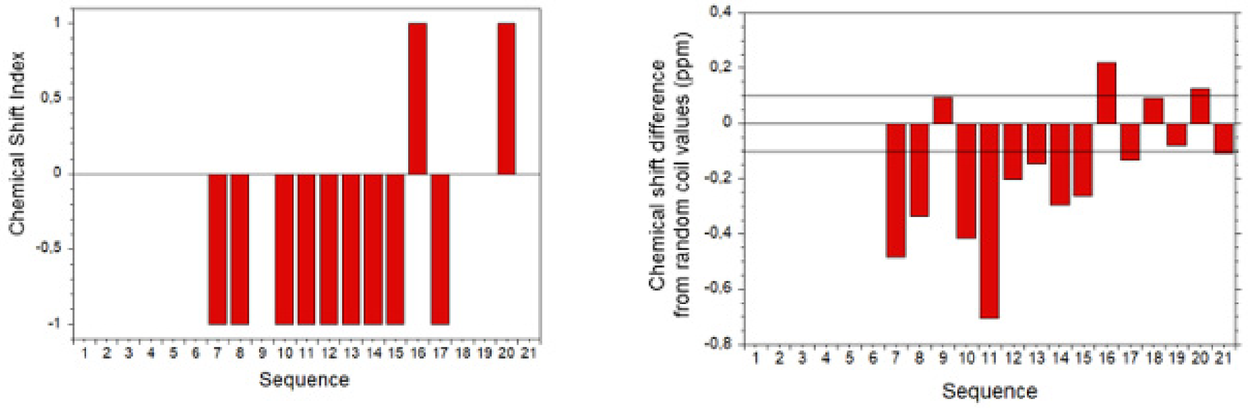

Figure S2. Chemical Shift Index of crabrolin21 in bicelles. Left panel: Chemical Shift Index of Hα protons vs. sequence; Right panel: chemical shift deviations of Hα protons from their respective random coil values.

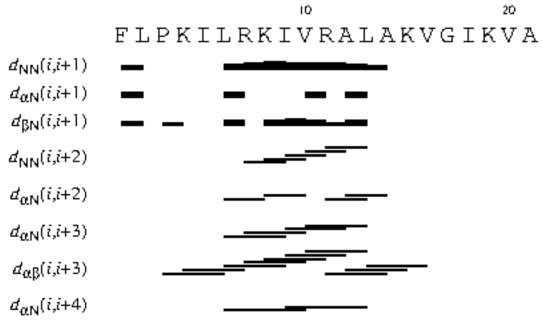

Figure S3. Observed NOEs for crabrolin21 in TFE. A plot of the short and medium-range upper distance limits against the sequence for crabrolin21 in TFE as derived from the NOESY spectrum at a mixing time of 150 ms.

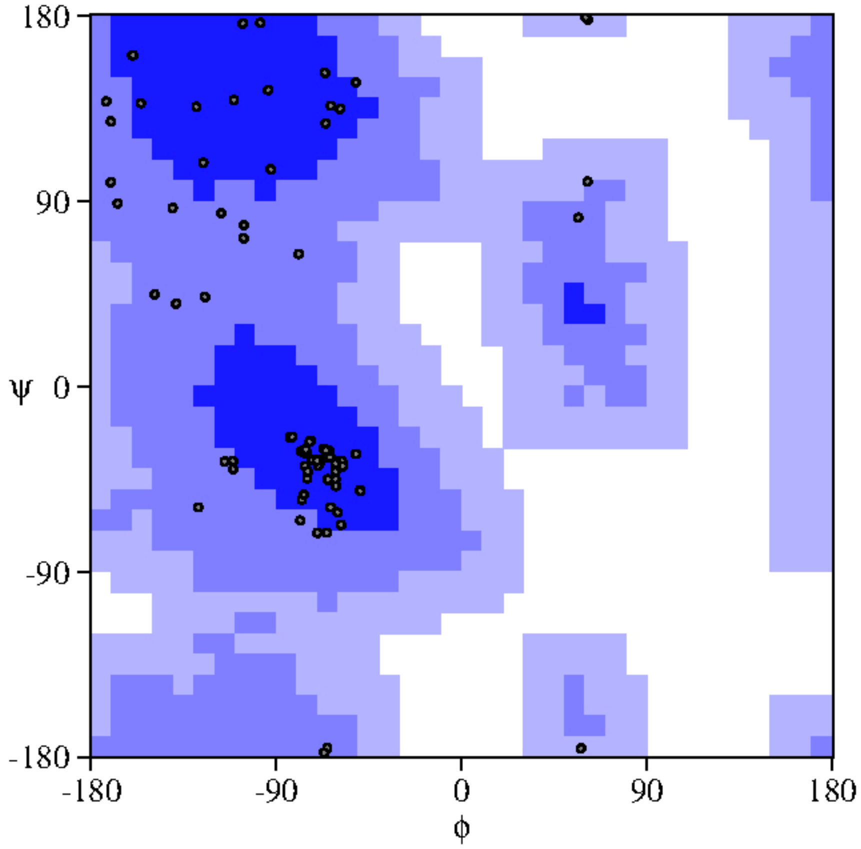

84.7% in most favored regions
13.5% in additionally allowed regions
1.8% in generously allowed regions
0.0% in disallowed regions

Figure S4. Ramachandran plot for crabrolin21 in TFE. Ramachandran plot of the structure family for crabrolin21 in TFE.

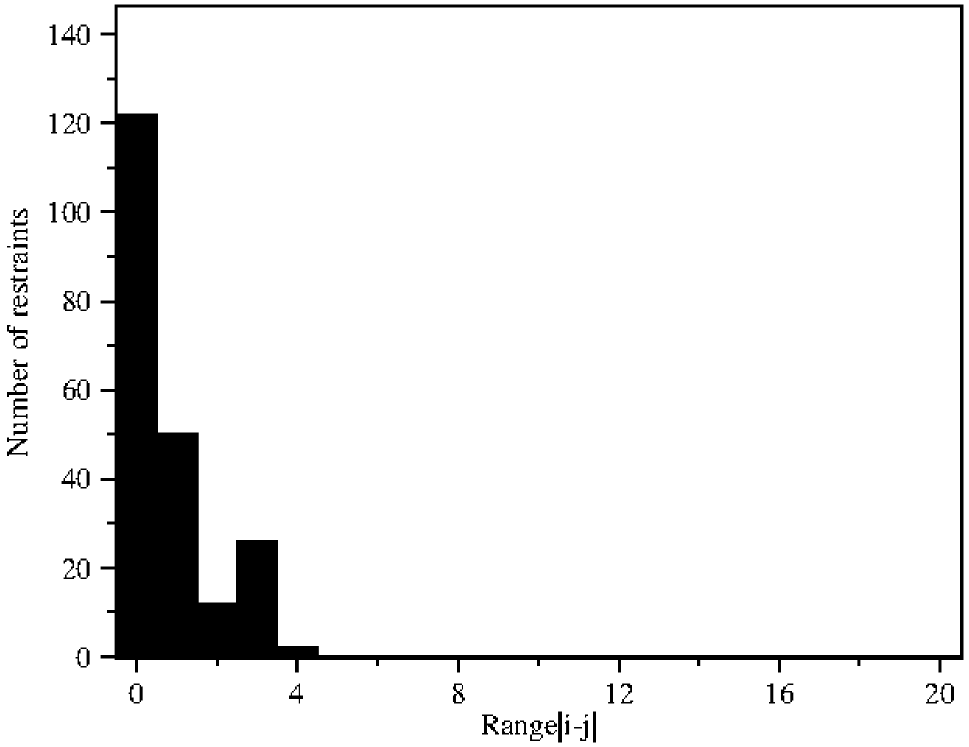
Number of restraints
140
120
100
80
60
40
20
0
0
4
8
12
16
20
Range|i-j|

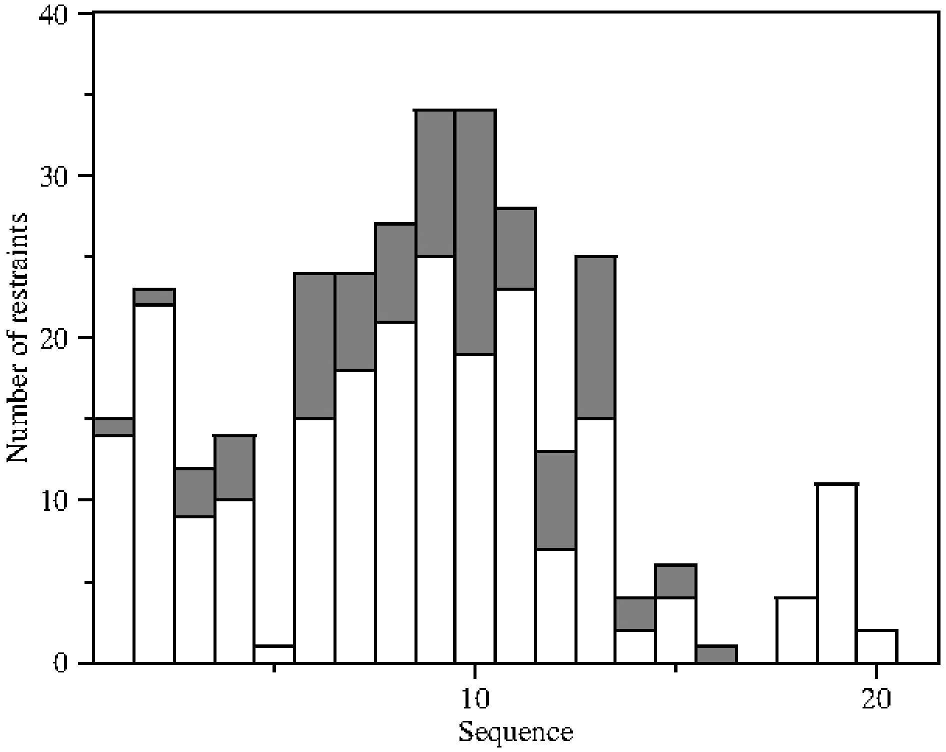
Number of restraints
40
30
20
10
0
10
20
Sequence

Figure S5. NMR distance constraints for crabrolin21 in TFE. Upper: Range distribution of distance constraints for crabrolin21 in TFE as derived from the NOESY spectrum at 150 ms mixing time. Lower: number of constraints observed for each residue of crabrolin21 in TFE as derived from the NOESY spectrum at 150 ms mixing time. In the plot, upper distance limits against the sequence are classified according to their range: white intra-residual constraints, and light grey sequential constraints.

Table 1. Summary of restraints and structure quality factors of the determined family of conformations for crabrolin21 in TFE.

	**crabrolin21 (10 Conformers)[a]**
Total number of meaningful NOE upper distance constraints [b]	212
Intraresidue NOEs	122
Sequential NOEs	50
Medium Range NOEs 1<(I-J)<5	40
**Average RMSD to the mean (Å)**	
backbone atoms (4-15)	0.19±0.08
residual CYANA Target Function (Å^2)	0.67 ±0.07
**Ramachandran Plot summary from Procheck[c]**	
Most favoured regions	97.5 (84.7)%
Additional allowed regions	2.5 (13.5)%
Generously allowed regions	0.0 (1.8)%
Disallowed regions	0.0%
**Ramachandran Plot statistics from Richardson's lab[c]**	
Most favoured regions	98.3 (87.4)%
Allowed regions	1.7 (11.6)%
Disallowed regions	0.0 (1.1)%
**Structure quality Factors-overall statistics [c]**	
Procheck G-factor Z-score (phi-psi)	0.42
Procheck (all)	-0.34
MolProbity Clashscore Z-score	1.49
**Close Contacts and Deviations from Ideal Geometry**	
Number of close contacts (within 1.6 Å for H atoms, 2.2 Å for heavy atoms	0
RMS deviation for bond angles	0.2°
RMS deviation for bond lengths	0.001 Å

[a] Structure calculations were performed with the program CYANA 3.97 [28]. A total of 100 random conformers were subjected to 10000 steps of a simulated annealing process. The mean value and the standard deviation are given.

[b] Number of meaningful constraints for each class.

[c] Selected regions ranges:4-16, data in parenthesis are obtained considering all 21 residues. Calculated using PSVS [34].

Ramachandran plot and NOEs restrains table are reported in **Figure S4** and **Figure S5** in Supplementary Material.
